# Exploitation of a Very Small Peptide Nucleic Acid as a New Inhibitor of miR-509-3p Involved in the Regulation of Cystic Fibrosis Disease-Gene Expression

**DOI:** 10.1155/2014/610718

**Published:** 2014-04-15

**Authors:** Felice Amato, Rossella Tomaiuolo, Fabrizia Nici, Nicola Borbone, Ausilia Elce, Bruno Catalanotti, Stefano D'Errico, Carmine Marco Morgillo, Giuseppe De Rosa, Laura Mayol, Gennaro Piccialli, Giorgia Oliviero, Giuseppe Castaldo

**Affiliations:** ^1^Dipartimento di Medicina Molecolare e Biotecnologie Mediche, Via Pansini 5, 80131 Napoli, Italy; ^2^CEINGE-Biotecnologie Avanzate, 80131 Napoli, Italy; ^3^Dipartimento di Farmacia, Università degli Studi di Napoli Federico II, Via D. Montesano 49, 80131 Napoli, Italy; ^4^Università Telematica Pegaso, 80143 Napoli, Italy

## Abstract

Computational techniques, and in particular molecular dynamics (MD) simulations, have been successfully used as a complementary technique to predict and analyse the structural behaviour of nucleic acids, including peptide nucleic acid- (PNA-) RNA hybrids. This study shows that a 7-base long PNA complementary to the seed region of miR-509-3p, one of the miRNAs involved in the posttranscriptional regulation of the CFTR disease-gene of Cystic Fibrosis, and bearing suitable functionalization at its N- and C-ends aimed at improving its resistance to nucleases and cellular uptake, is able to revert the expression of the luciferase gene containing the 3′UTR of the gene in A549 human lung cancer cells, in agreement with the MD results that pointed at the formation of a stable RNA/PNA heteroduplex notwithstanding the short sequence of the latter. The here reported results widen the interest towards the use of small PNAs as effective anti-miRNA agents.

## 1. Introduction

In the last twelve years a new group of endogenous, small, noncoding fragments of RNA, 18–25 nucleotides in length, named microRNAs (miRNAs) emerged for its ability to suppress the gene expression at posttranscriptional level [1, 2]. To date more than 1,400 miRNAs have been identified. MicroRNAs regulate the gene expression by annealing with the complementary mRNAs, thus preventing their translation or inducing their degradation [3, 4]. Although miRNAs usually recognize the 3′UTR many of them are capable of binding the 5′UTR or even coding regions of target mRNAs. Due to the small number of constituting nucleobases, each miRNA can recognize one or many mRNAs and each mRNA can be the target of many miRNAs. The result of this network of interactions is the coregulatory role of miRNAs on the translation/degradation of one or more mRNAs [5]. Despite the potential occurrence of off-target effects, it is emerging that the modulation of specific miRNAs represents a new approach to achieve the control of gene expression. Potential applications of miRNA inhibitors (antimiR) range from diagnostics to regulation of important proteins involved in numerous cancers [6]. A number of human diseases have been associated with a deregulation of specific miRNAs [7–12]. Among these is the genetic disease Cystic Fibrosis (CF). CF is the most common lethal genetic disorder among Caucasians with one in every 3,000 newborns affected. CF is due to mutations in the CFTR gene encoding the CFTR chloride channel expressed by most epithelial cells [13]. The CF phenotype typically includes the altered sweat test, pancreatic insufficiency, and pulmonary infections that gradually lead to respiratory insufficiency. To date more than 1,900 mutations of CF gene have been described, and a set of miRNAs inhibiting the CFTR expression at the posttranscriptional level has been described [14]. Furthermore, our group has shown that mutations in the 3′UTR of the CFTR gene may have a pathogenic effect by enhancing the affinity for the miR-509-3p miRNA [15].

The approaches to downregulate a specific miRNA essentially use oligonucleotide (ON) analogues which being complementary to miRNAs are able to reduce or inhibit their activity. For this purpose a number of ribose modified ONs, usually bearing a phosphorothioate backbone, have recently been used. Interesting results have been obtained by using 2′-O-methyl-ribonucleotides [16, 17] and other 2′-modified ONs [18, 19]. In addition, locked nucleic acids (LNAs) have shown interesting activity [20, 21] especially when used in combination with unmodified DNA monomers. Recently, several studies have demonstrated that the DNA mimics named peptide nucleic acids (PNAs) can be effectively used as anti-miRNA [22–24]. In the PNAs a 2-aminoethyl-glycine polymer replaces the ribose-phosphate DNA backbone [25]. PNA molecules are resistant to protease and nuclease degradation and recognize with a high affinity complementary fragments of DNA or RNA [26]. Many studies have been performed on the binding capability of PNAs and on the topological way in which they can recognize nucleic acids in single strand, duplex, or quadruplex arrangements to form heteroduplex, heterotriplex, and heteroquadruplex complexes [27–31] or to act as quadruplex ligands, respectively [32, 33]. The anti-miRNA activity of a PNA can occur in the nucleus by targeting the pre-miRNA or in the cytoplasm by binding the pre-miRNA and/or the mature miRNA [17]. In both cases it is necessary that the PNA can pass through the cell membrane and also through the nuclear membrane for the former case. The main drawback in the use of PNAs as intracellular probes lies in the poor water solubility when their length exceeds the 12–14 bases. Furthermore, the cellular uptake behaviour of a PNA is not easily predictable because it is mostly dependent on the PNA base composition and the overall lipophilicity. Recent studies report on the feasibility of a miRNA regulation approach by using unmodified PNAs and PNAs conjugated with peptides or hydrophilic groups [34, 35].

PNAs having a poly-lysine tail display increased water solubility and cellular uptake [23, 24]. In addition, negatively charged PNAs can be obtained by synthesizing PNA-DNA hybrid strands or by attaching negative groups to the PNA monomers [34, 36]. In the last case, cationic lipids can be used as transfection reagents.

We recently reported that some anionic PNAs, synthesized by our group, are a potential treatment for CF by targeting the miR-509-3p involved in the regulation of CF disease-gene expression [37]. In that study we synthesized a 14-base long PNA fully complementary to the 5′-end of miR-509-3p and carrying a tetrapeptide tail containing two serine phosphates at its C-terminus and a fluorescein group at its N-terminus (PNA1, Table 1). We demonstrated, by* in vitro* studies on A549 cell lines, that the serine phosphate tail represents a suitable conjugation to improve both the water solubility and the cellular uptake of a PNA molecule. Hybridization studies on PNA1 in the presence of miR-509-3p, performed by UV and CD spectroscopies and by electrophoretic mobility shift assay (EMSA), prove that the anionic peptide tail does not hamper the formation of the miR-509-3p/PNA1 heteroduplex. Finally, by reverting the expression of the luciferase gene containing the 3′UTR of the CFTR gene, we also demonstrated that PNA1 is able to recognize miR-509-3p in A549 cells. In continuing our studies on the Cystic Fibrosis and on the control of the related miR-509-3p miRNA, we decided to test the capability of the short 7-mer PNA2 (Table 1), bearing the same functionalization of PNA1 and complementary to the seed region of miR-509-3p, to bind this miRNA. Our interest towards shorter PNA anti-miRNAs was triggered by the consideration that the synthesis of longer PNAs (14–16 bases long) is an expensive and not an easily achievable task, especially when the PNA is conjugated to peptide tails and/or labelled at both ends. In addition, a recent study has reported that a very short LNA (8 bases long) was able to recognize and silence a family of miRNAs with no off-target effects [38]. Furthermore, experimental and computational evidence for different types of miRNA target sites demonstrated that probes with as few as seven base pairs of complementarity to the 5′-end of miRNAs are sufficient to confer regulation* in vivo* and are used in biologically relevant targets [5, 39]. The synthesis of PNA2 was preceded by a molecular modelling study aimed at evaluating the structural behaviour of the goal seven bases long miR-509-3p/PNA2 heteroduplex in comparison with that of the longer miR-509-3p/PNA1 heteroduplex. The stability and the structure of the miR-509-3p/PNA2 duplex were evaluated by molecular modelling and by UV, CD, and EMSA analyses. We here anticipate that PNA2, notwithstanding its reduced length, was still able to recognize miR-509-3p in A549 cells where it reverted the expression of the luciferase gene containing the 3′UTR of the CFTR gene.

## 2. Materials and Methods

### 2.1. Synthesis of miR-509-3p and PNAs (Table 1)

The miR-509-3p mimic (2′-OMe modified) was synthesized and purified by the oligonucleotide synthesis facility at CEINGE-Biotecnologie Avanzate (Naples, Italy). The 4-methyl-benzhydrylamine-resin (MBHA resin, 0.4 mmol/g), all Fmoc/Boc protected monomers, and the 2-(2-(fluorenylmethoxycarbonylamino)ethoxy) ethoxyacetic (AEEA) spacer-linker were purchased from Link Technologies. Fmoc-L-Ser(PO(OBzl)OH)-OH building block, 2-(1-H-benzotriazol-1-yl)-1,1,3,3-tetramethyluronium hexafluorophosphate (HBTU), 2-(1-H-7-azabenzotriazole-1-yl)-1,1,3,3-tetramethyluronium hexafluorophosphate (HATU), and 1-hydroxybenzotriazole (HOBt) were purchased from Novabiochem. The following abbreviations are used: trifluoroacetic acid (TFA), dimethylformamide (DMF), dichloromethane (DCM), N,N-diisopropylethylamine (DIPEA), N-methyl-pyrrolidone (NMP), 1,8-diazabicyclo(5,4,0)undec-7-ene (DBU), and 2-(6-hydroxy-3-oxo-3H-xanthen-9-yl)-5-isothiocyanate-benzoic acid (FITC).

PNA2 and PNA3 were synthesized using the Fmoc-solid-phase strategy. The MBHA resin (50 mg, 0.02 mmol), after swelling in DCM (30 min) and DMF washings, was treated with a solution of 20% piperidine (2 mL) in DMF for 10 min. After washings in DMF, the resin was reacted with Fmoc-Gly (5 eq., in NMP 0.25 M), HATU (3.6 eq. in DMF 0.2 M), and DIPEA (5 eq.)/lutidine (6 eq.) for 1 h at room temperature. During the peptide and PNA synthesis the Fmoc group was removed by a treatment with a 5% DBU in DMF solution (5 min). In the case of Fmoc-Ser amino acids the basic treatment was prolonged (20 min). Couplings of Fmoc-L-Ser(PO(OBzl)OH)-OH were achieved using the following conditions: Fmoc-Ser monomer (8 eq. in NMP 0.4 M), HATU (8 eq. in DMF 0.4 M), and DIPEA (8 eq.)/lutidine (12 eq.) for 15 h at room temperature. PNA monomers and AEEA-COOH linker were reacted using the following conditions: monomer building block (8 eq. in NMP 0.4 M), HATU (8 eq. in DMF 0.4 M), and DIPEA (8 eq.)/lutidine (12 eq.) for 4 h at room temperature.

For the coupling with the fluorescent group the FITC monomer (5 eq., 0.2 M) was dissolved in DMF/DIPEA (2.5 : 97.5 v/v) and the solution was added to the resin, which was gently shaken in the dark for 15 h. The resin was finally treated with TFA/anisole/ethanedithiol (9 : 0.5 : 0.5; v/v/v) for 3.5 h and the products were precipitated with cold diethyl ether. The precipitates were recovered by centrifugation and following two washings with diethyl ether were dissolved in water and lyophilized. The PNA2 and PNA3 were obtained with a 48–50% overall yield (94-95% medium yield for each coupling).

The purifications were performed by HPLC using a RP-18 column (Merck, RT 250–10 5 *μ*m) eluted with a linear gradient from 10% to 90% of eluent B in eluent A in 30 min. Eluent A: 0.1% TFA in water; eluent B: 0.1% TFA in acetonitrile. For these purifications the UV/VIS detector was set at 495 nm corresponding to the maximum of absorption of FITC. The collected yellow fractions were lyophilized and stored at −20°C in the dark.

The structures of PNA2 and PNA3 were confirmed by MALDI-TOF mass spectrometry on a Bruker Autoflex I instrument using *α*-cyano-4-hydroxycinnamic acid, 10 mg/mL in acetonitrile-3% aqueous TFA (1 : 1, v/v) as the matrix.

 PNA2 m/z calculated 2812, found 2813 [M + H]^+^.
 PNA3 m/z calculated 2864, found 2865 [M + H]^+^.

### 2.2. Molecular Modelling

The initial structures of the heteroduplexes formed by miR-509-3p with PNA1 and PNA2 were built by using the NMR structure of the 6-mer RNA(GAGUUC)/PNA(GAACTC) heteroduplex (PDB ID = 176D) [43]. Starting from the lowest energy NMR structure, one nucleotide was added aligning a duplicate of the reference structure on the PNA backbone. Once the 7-mer heteroduplex was obtained, the bases were mutated to match the PNA2 sequence. Watson-Crick canonical pairs were then refined using distance restraints on the first seven bases of miR-509-3p/PNA2 heteroduplex. The same procedure was used to build the miR-509-3p/PNA1 heteroduplex, starting from the refined structure of miR509-3P/PNA2 heteroduplex.

The equilibration of the systems and production of MD simulations were performed using the Amber 12 suite of programs [44, 45]. The Leap module of Ambertools13 was used to create parameter and topology files for the MD simulations using the ff99SB force field for RNA and standard amino acids [44, 45]. For PNAs parameterization we used the Sanders et al. force field for PNA [46] downloaded from the RESP and ESP charge database (R.E.DD.B. http://q4md-forcefieldtools.org/REDDB Project ID = F93) [47], whereas the parameters for serine phosphate were taken from reference [48]. TIP3P water molecules were added with a minimum spacing of 10.0 Å from the box edges to the RNA:PNA molecules and Na^+^ counterions were added to each system to reach the neutralization of the system.

The geometry of the system was minimized in four steps as follows: (1) optimization of hydrogen atoms (500 steps of steepest descent and 4,500 steps of conjugate gradient); (2) optimization water molecules and counterions (2,000 steps of steepest descent and 8,000 steps of conjugate gradient); (3) further optimization of hydrogen atoms, water molecules, and counterions (3,500 steps of steepest descent and 11,500 steps of conjugate gradient); (4) final optimization of the whole system (2,500 steps of steepest descent and 8,500 steps of conjugate gradient). Thermalization of the system was performed in four steps of 60 ps, increasing the temperature from 10 to 298 K. Concomitantly, interstrand distance restraints were applied to the RNA:PNA heteroduplex to preserve all base pairs canonical Watson-Crick bond, allowing 0.1 Å movement from the equilibrium bond distance (either closer or farther). Thus, the force constant applied during thermalization was set to 32 kcal mol^−1^ Å^−2^ and was gradually reduced in the next step to 10 kcal mol^−1^ Å^−2^ and subsequently decreased by increments of 5 kcal mol^−1^ Å^−2^ in the next stages. Then, an additional step of 250 ps was performed in order to equilibrate the system density at constant pressure (1 bar) and temperature (298 K). Finally, an extended trajectory covering was run using a time step of 2 fs. SHAKE was used for those bonds containing hydrogen atoms in conjunction with periodic boundary conditions at constant pressure and temperature, particle mesh Ewald was used for the treatment of long range electrostatic interactions, and a cutoff of 9 Å was used for nonbonded interactions.

All production simulations were repeated in triplicate with random seeding for initial velocities and extended to 20 ns. In order to further assay the stability of the RNA:PNA heteroduplexes, we extended one run of PNA2 up to 50 ns, for a total simulation time of 90 ns for PNA2 and 60 ns for PNA1. The structural features were determined using the Curves+ software package [40], and visualization of trajectories was performed in VMD [41], while the trajectory analyses were performed using Ambertools13.

### 2.3. Preparation of miRNA/PNA Heteroduplexes (Annealing)

The miR-509-3p/PNA heteroduplexes (1 : 1.5 or 1 : 5) were formed by heating the mixture of the samples dissolved in 100 mM KCl, 10 mM K_2_HPO_4_, at 90°C for 5 min and slowly cooling at room temperature for 12 h. The amount of each PNA sample was estimated by quantitative UV at 80°C using the following molar extinction coefficients: PNA1 *ε* = 149.6 mL *μ*mol^−1 ^cm^−1^, PNA2 *ε* = 69.7 mL *μ*mol^−1 ^cm^−1^, PNA3 *ε* = 61.6 mL *μ*mol^−1 ^cm^−1^, and miR-509-3p *ε* = 205.0 mL *μ*mol^−1 ^cm^−1^.

### 2.4. UV and UV Melting Studies

The UV spectra were recorded on a Jasco V-530 UV spectrophotometer equipped with a Peltier-type temperature control system (model PTC-348WI). Thermal denaturation experiments were carried out in the temperature range 5–90°C by monitoring the absorbance at 260 nm at the heating rate of 0.5°C/min. The apparent Tm was estimated from the maximum in the first derivative of the melting profile.

### 2.5. CD Studies

CD spectra were recorded with a Jasco J-715 spectropolarimeter equipped with a Peltier Thermostat Jasco ETC-505T using 0.1 cm path length cuvettes and calibrated with an aqueous solution of 0.06% d-10-(1)-camphorsulfonic acid at 290 nm. The molar ellipticity [Θ] (deg cm^2^ dmol^−1^) was calculated from the following equation: [Θ] = [Θ]obs/10  *l* *C*, where [Θ]obs is the ellipticity (mdeg), *C* is the oligonucleotide molar concentration, and *l* is the optical path length of the cell (cm). CD measurements (220–320 nm) were carried out at a scan rate of 100 nm/min with a 2 nm bandwidth. The concentration of miR-509-3p/PNA2 and miR-509-3p was 1.0 × 10^−5 ^M. The spectra were signal-averaged over at least three scans and baseline was corrected by subtracting the buffer spectrum.

### 2.6. Cell Line, Construct, and Transfections

A549 human lung carcinoma cells were purchased from ATCC (Manassas, USA). Cells were maintained in Dulbecco's modified Eagle's medium (Gibco Invitrogen, USA) with 10% heat inactivated fetal bovine serum (HyClone, USA) without the addition of antibiotics. Luciferase construct bearing the 3′UTR of CFTR gene [15] was used as miR-509-3p sensitive. Transfection of A549 cells with miRNA-mimics (Qiagen, Germany, EU) or PNA was performed with Attractene Transfection Reagent (Qiagen) as previously reported [37]. Briefly, cells seeded in 96-well plates were cotransfected with the luciferase reporter constructs and the miR-509-3p mimic. 24 h after, the cells were transfected with anti-miR-509-3p PNA. The transfection efficiency (*≈*80%) was assessed by measuring the percentage of fluorescent cells relative to the total number of cells. The luciferase activity level was measured 24 h after transfection using the Dual-Glo Luciferase Assay System (Promega Corporation). The relative reporter activity was obtained by normalization to the Renilla luciferase activity.

### 2.7. Electrophoretic Mobility Shift Assay

The miR-509-3p mimic (2′OMe-modified) was synthesized by the oligonucleotide synthesis facility at CEINGE-Biotecnologie Avanzate (Naples, Italy). As previously reported [37], the miRNA and PNA were annealed in 1X NEBuffer 2 (50 mM NaCl, 10 mM Tris-HCl, 10 mM MgCl_2_, 1 mM DTT, and pH 7.9 at 25°C) for 2 h at room temperature. All the reactions were loaded into 20% polyacrylamide gels in 0.5X Tris-Borate-EDTA (TBE) buffer and run at 140 V for 3 h. The fluorescence signal was acquired placing the wet gel directly on the plate of the Typhoon 8600 scanner.

## 3. Results and Discussion

With the aim of evaluating the feasibility of our hypothesis of shortening the PNA1 molecule to achieve a more synthetically affordable PNA targeted against miR-509-3p that preserves the hybridization properties of the parent PNA1, PNA2 was designed by deleting all the PNA1 bases that were not complementary to the “seed region” of miR-509-3p (i.e. the first seven bases at its 5′end, considered the most important target to achieve the anti-miRNA activity). As previously done for PNA1 [37], to improve the water solubility and the cellular uptake of PNA2 we decided to add the negatively charged tetrapeptide G-S(P)-S(P)-G at the C-end, whereas the fluorescent AEEA linker-FITC was added at the N-end to assess the cellular localization of PNA2. Before proceeding to the in-lab synthesis of PNA2, we estimated the stability and the conformational features of the goal miR-509-3p/PNA2 heteroduplex by means of computational techniques and compared the results with those of the correspondent heteroduplex formed with the PNA1. Computational techniques, and in particular molecular dynamics (MD) simulations, have been successfully used as complementary technique to predict and analyse the structural behaviour of nucleic acids, including PNA-RNA hybrids [46, 49, 50].

### 3.1. Molecular Dynamics Simulations

The miR-509-3p/PNA2 and miR-509-3p/PNA1 heteroduplexes were built starting from the NMR structure of the RNA(GAGUUC)/PNA(GAACTC) duplex (PDB-ID 176D) [43] as described in Materials and Methods. Each system was firstly analysed by means of three runs of 20 ns molecular dynamics in order to better sample the conformational behaviour of the complexes. Secondly, in order to further assay the stability of the miR-509-3p/PNA2 duplex, we extended one run of miR-509-3p/PNA2 up to 50 ns, for a total simulation time of 90 ns for miR-509-3p/PNA2 and 60 ns for miR-509-3p/PNA1.

**Figure 1:**
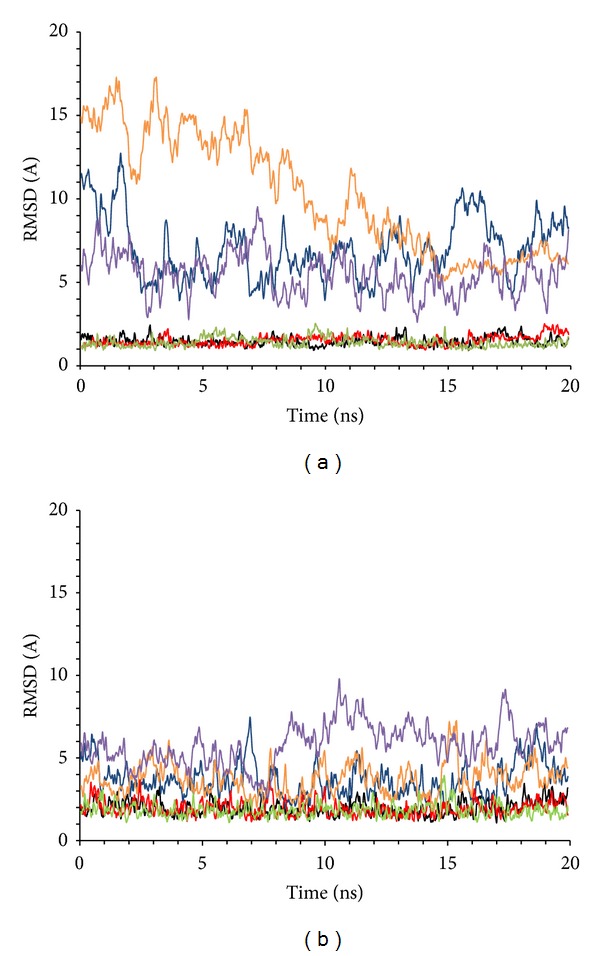
Root-mean-square deviation in the three MD runs on miR-509-3p/PNA2 (a) and miR-509-3p/PNA1 (b) heteroduplexes. Superimpositions were made on the MD-averaged structures for each trajectory taking into account the whole structure (blue, orange, and purple) or only the duplex region (black, red, and green).

The macroscopic properties of the systems, such as temperature, pressure, volume, density, and energy, were fairly constant during the whole simulation for both of the systems (data not shown). As expected, the analysis of the RMSD in the trajectories of miR-509-3p/PNA2 and miR-509-3p/PNA1 complexes showed high flexibility of the single stranded miRNA segment (Figure 1). On the contrary, the behaviour of the region of miR-509-3p hybridized with PNAs was characterized by low RMSD values and low fluctuations, thus indicating the presence of a stable secondary structure (Figure 1).

**Figure 2:**
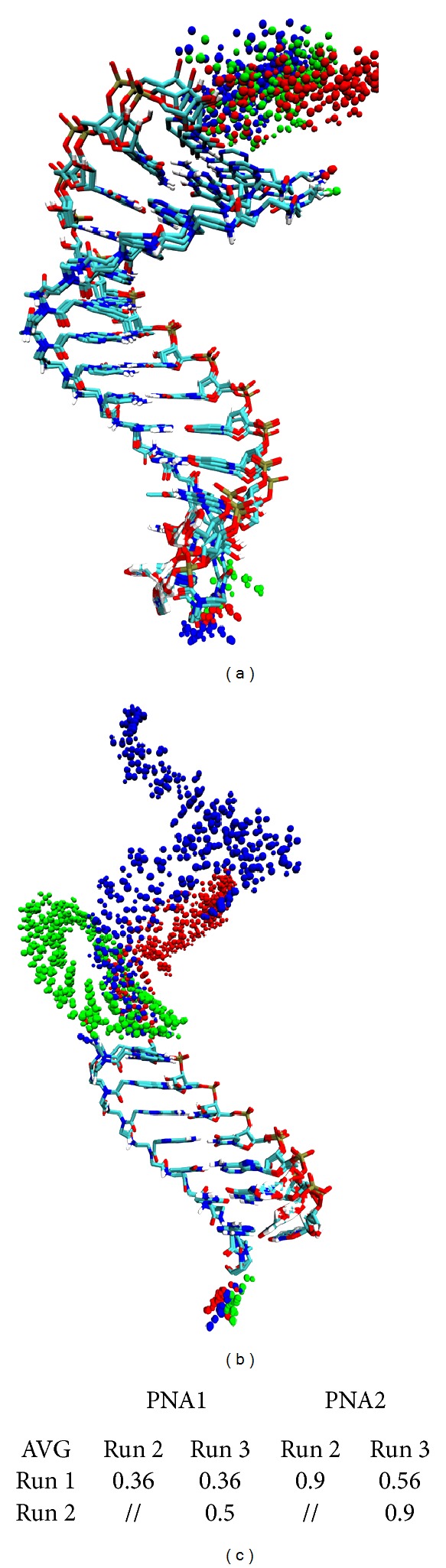
Superimposition of the MD average structures of miR-509-3p/PNA1 complex (a) and of miR-509-3p/PNA2 complex (b). Duplexes regions are represented in licorice coloured by atom type (carbon in cyan, oxygen in red, nitrogen in blue, phosphate in brown, and hydrogen in white). The miR-509-3p single strand regions are represented in spheres coloured by MD run (blue, run 1; green, run 2; red, run 3). (c) RMSD in Å, calculated on phosphates in the duplexes regions, among the average structures of the MD runs.

Moreover, the comparison of the average structures obtained from each trajectory (Figure 2) revealed the convergence of the trajectories as shown by the low RMSD values in the duplex region of both complexes with PNA1 and PNA2 (>0.5 Å and >0.9 Å, resp.).

The analysis of the helicoidal parameters and torsion angles (Table 2) demonstrated that both miRNA/PNA duplexes could be described as A-type double helix with few noticeable deviations from the canonical structure. In particular, the lower step-averaged twist values reported in Table 2 indicated a slight unwinding of the RNA/PNA helices with respect to canonical A-RNA structures, in agreement with what was previously observed in other MD simulations of RNA/PNA duplexes [43, 49]. The lower roll and tilt values observed in the MD run pointed at an expansion of the major groove. Finally, the analysis of torsion angles reported in Table 2 highlighted the strong similarity between the two duplexes. Taken together, the MD results indicated that both heteroduplexes assumed a conformation resembling the canonical A-type RNA helix rather than the experimentally determined NMR structure. Moreover, the torsion angles of RNA segments in miR-509-3p/PNA2 and miR-509-3p/PNA1 duplexes showed values very similar to those adopted by miR20a in the 4F3T crystal structure [42] suggesting that PNA2 and PNA1 could easily interact with the AGO-miRNA complex, not requiring any conformational adaptations. On the basis of the positive indications coming from the MD studies, we synthesised the PNA2 molecule and studied its ability to recognize the 2′-OMe mimic of miR-509-3p by CD, UV, and EMSA studies and evaluated its ability to restore the expression of the luciferase gene containing the 3′UTR of the CFTR gene in the presence of miR-509-3p.

### 3.2. Synthesis of PNA2 and PNA3

PNA2 and PNA3, chosen as the negative control and bearing the same functionalization of PNA2, were synthesized using the standard Fmoc-solid-phase strategy on the Rink-amide resin following the previously reported synthetic approach [37]. The sequences and the complete structures of PNA2 and PNA3 are shown in Table 1.

### 3.3. UV and UV Melting Studies

**Figure 3:**
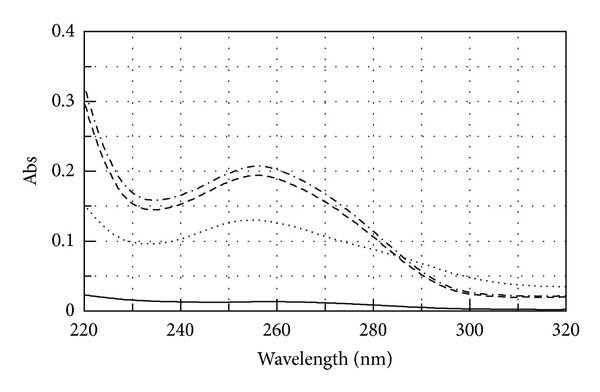
UV spectra of miR-509-3p (dashed line), PNA2 (solid line), miR-509-3p/PNA2 mixture (1 : 1.5) (dotted line), and the arithmetical sum (dashed-dotted line).

**Figure 4:**
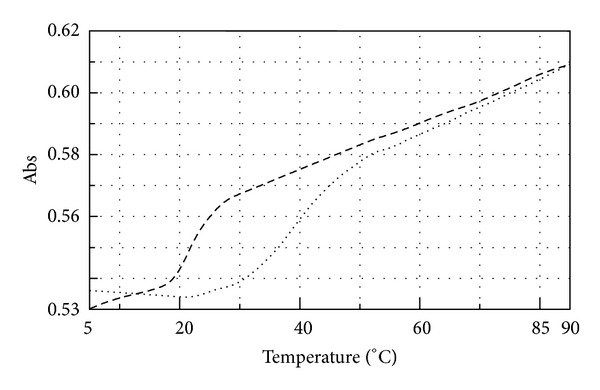
UV melting profile of miR-509-3p alone (dashed line) and miR-509-3p/PNA2 mixture (1 : 1.5 dotted line).

The miR-509-3p/PNA2 complex, prepared as described in Materials and Methods, was analysed by UV spectroscopy in the temperature range of 25–90°C. The data showed for the miR-509-3p/PNA2 complex a lower value of absorbance than the arithmetic sum of each component alone (Figure 3), thus evidencing that heteroduplex stacking interactions between the miRNA strand and the PNA2 had occurred. The UV melting experiments performed on the miR-509-3p/PNA2 mixture (1 : 1.5 ratio) showed a sigmoidal profile, which was indicative for the heteroduplex/single strands transition (Figure 4). The calculated apparent melting temperature of the miRNA/PNA2 heteroduplex was 40°C. The UV melting profile of the sole PNA2 did not show any significant variation in the A_260_ value in 10–70°C (data not shown), whereas, the UV melting of the sole miR-509-3p, in the same experimental conditions, showed a sigmoidal profile with an apparent melting temperature of 26°C, which could be attributed to the melting of poorly stable secondary structures of the miRNA. This data suggests that PNA2 is able to form a complex with miR-509-3p provided with the thermal stability required for* in vivo* experiments.

### 3.4. Circular Dichroism Spectra Analyses

**Figure 5:**
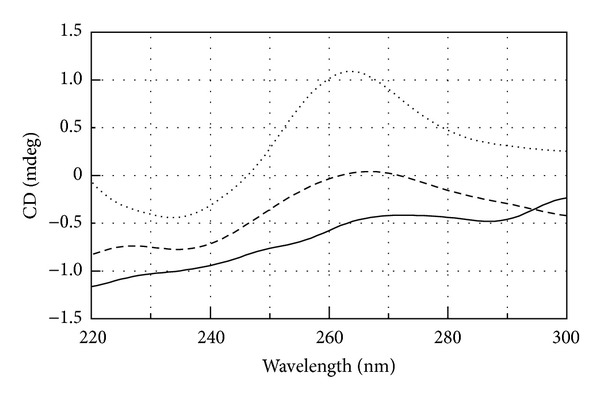
CD spectra of miR-509-3p alone (dashed line), PNA2 alone (solid line), and miR-509-3p/PNA2 mixture (1 : 1.5) (dotted line).

To further confirm the formation of the miR-509-3p/PNA2 heteroduplex complex, circular dichroism (CD) spectra were registered for the miR-509-3p, PNA2, and their 1 : 1.5 mixture after the annealing procedure (Figure 5). In particular, the miR-509-3p/PNA2 mixture showed the typical CD profile of antiparallel RNA/PNA heteroduplexes, characterized by maxima at around 260 and 220 nm and minima at around 235 and 196 nm, thus confirming the capability of the PNA2 to form a heteroduplex with the miR-509-3p miRNA.

### 3.5. EMSA Results

**Figure 6:**
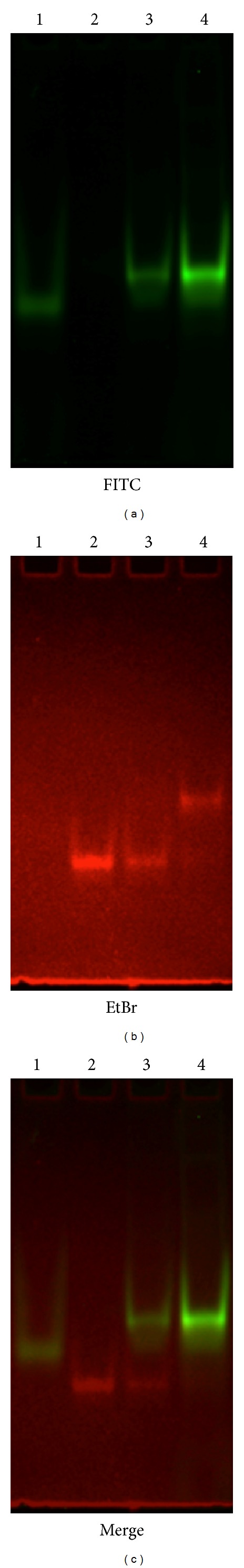
EMSA of PNA2 alone (lanes 1), miR-509-3p alone (lanes 2), miR-509-3p/PNA2 1 : 1.5 (lanes 3), and miR-509-3p/PNA2 1 : 5 (lanes 4) visualized by FITC (a) or EtBr (b) staining. The superimposition of FITC and EtBr stained gels is shown in (c).

The recognition phenomena between the miR-509-3p and the FITC-labelled PNA2 were also studied by electrophoretic mobility shift assay (Figure 6(a)). To allow the visualization of miR-509-3p alone, the gel was also visualized after the EtBr staining (Figure 6(b)). The superimposition of FITC and EtBr stained gels is shown in Figure 6(c). The electrophoretic mobility of PNA2 alone (lanes 1) was slower than that of miR-509-3p alone (lane 2). When miR-509-3p and PNA2 were mixed in the molar ratios of 1 : 1.5 and 1 : 5 (lanes 3 and 4, resp.) we observed the appearance of a new band, corresponding to the miR-509-3p/PNA2 complex, which was upshifted relative to the bands of the two components alone. The formation of the miR-509-3p/PNA2 complex was further confirmed by the disappearance of the band of the free miR-509-3p in the miRNA/PNA2 1 : 5 complex (lane 4, Figures 6(b) and 6(c)). The EMSA data further corroborated the CD evidence about the ability of PNA2 to form a stable complex in the presence of miR-509-3p miRNA.

### 3.6. Biological Activity

**Figure 7:**
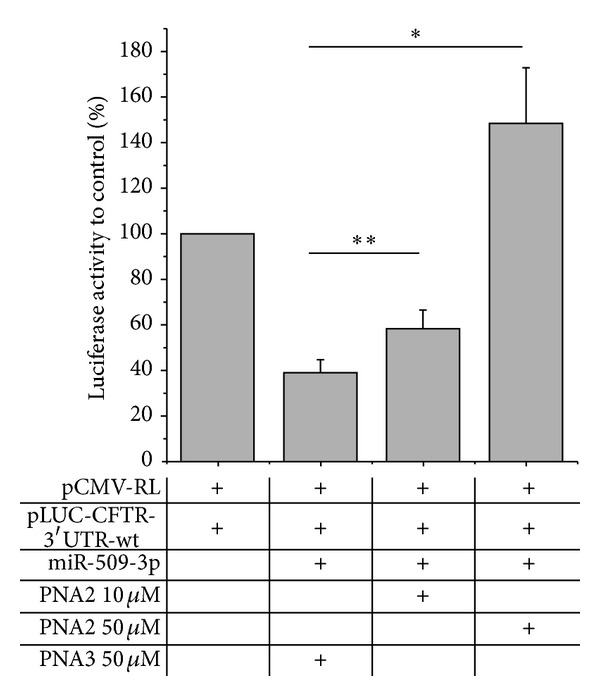
Inhibition of miR-509-3p effect by PNA2. Different doses of PNA2 were transfected in A549 cells. A significant inhibition of miR-509-3p was observed using PNA2 in a dose-dependent manner. *P values <0.02, **P values <0.002. The 7-mer poly-thymine PNA3 had no effect on miR-509-3p.

**Figure 8:**
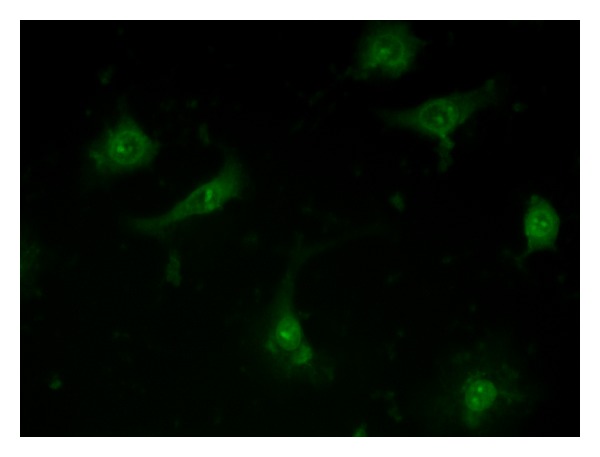
Representative uptake of FITC-labelled PNA2 by A549 cells.

Once the ability of PNA2 to form a stable heteroduplex with miR-509-3p was demonstrated, we examined its potential of being a miR-509-3p inhibitor in a biological context. For this purpose we tested the ability of PNA2 to revert the reduction of luciferase activity induced by the transfection of the 2′-OMe mimic of miR-509-3p in A549 cells. As shown in Figure 7 the transfection of PNA2, but not of PNA3, was able to rescue the luciferase activity in a dose-dependent manner. In this experiment A549 cells were first transfected with the pLuc-CFTR-3′UTR plasmid (a reporter luciferase construct sensitive to the miR-509-3p mimic action due to the presence of the 3′UTR of the CFTR gene) and with miR-509-3p miRNA. As expected, the transfection of the miR-509-3p reduced the luciferase activity down to 40%. The luciferase activity was rescued after the transfection of the PNA2 in a dose-depended manner. In these experiments the commercially available Attractene cationic lipid transfection reagent was used. The fluorescent microscopy image of the A549 cells taken 24 h after the transfection with PNA2 (Figure 8) confirmed the PNA2 uptake by the cells.

## 4. Conclusions

Previously, we showed that the activity of the miR-509-3p miRNA, one of miRNAs involved in the posttranscriptional regulation of CFTR gene of CF and CF-RD, could be inhibited through the use of the 14-mer PNA1 fully complementary to the first fourteen bases of miR-509-3p [37]. With this study, we demonstrate that the activity of miR-509-3p can be inhibited even with the use of a PNA as short as seven bases long targeting exclusively the seed region of the miRNA. This finding, probably due to the higher affinity of PNAs over RNAs towards the complementary RNA strand, further widens the interest towards the use of peptide nucleic acids as effective anti-miRNA agents, considering the number of advantages in terms of cost and time saving in the synthesis of the PNAs or for what attains their cellular uptake.

## Figures and Tables

**Table 1:** Structures and sequences of miR-509-3p and PNA1–3. PNA sequences are written from C- to N-terminus.

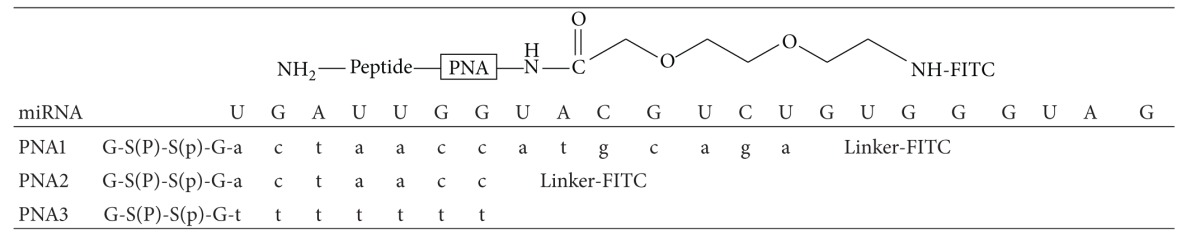

**Table:** (a)

Helicoidal parameters							
Duplex name	Twist (°)	Roll (°)	Tilt (°)	Inclination (°)	H.Ris (°)	H.Twi (°)	Groove width (Å)
PNA:RNA_NMR^a^	30.1 (4)	4.9 (4.3)	1.0 (3.3)	9.0 (2.7)	2.98 (0.2)	30.6 (4.2)	6.1 (0.5)
PNA:RNA_MD^b^	23.7; 23.2						
A-RNA^c^	32	12	2.8	15.8	3.3		3.8
PNA2 AVG tot	24.5 (1.1)	6.5 (1.6)	1 (0.9)	14.2 (0.6)	2.79 (0.10)	25.3 (1.1)	6.8 (0.6)
PNA1 AVG tot	24.9 (2.8)	5.7 (2.2)	0.9 (1.3)	13 (1.1)	2.77 (0.16)	25.5 (2.9)	6.8 (0.5)

**Table:** (b)

Torsional PNA angles (in degrees)							
	N4–C5	C5–C	C–N1	C2–C3	C3–N4	N4–C7	*χ*
PNA:RNA_NMR^a^	−84.9	80	105.7	66	−100.1	9.1	50.6
PNA2	**−79.9**	**125.2**	**78.6**	**70.4**	**−103**	**−2.9**	**78**
PNA1	**−81.3**	**129**	**131.5**	**69.5**	**−103.3**	**−2.1**	**78.5**

**Table:** (c)

Torsional RNA angles (in degrees)							
	*α*	*β*	*γ*	*δ*	*ε*	*ζ*	*χ*
PNA:RNA_NMR^a^	−68.4	111.75	58.4	78.5	−148.7	−72.5	−104.5
A-RNA^c^	−52	175	42	79	−146	−75	−157
miR20a^d^	−99.1	162.6	73.1	88.7	−138	−119.4	−147.6
PNA2 AVG tot	−84.4	172.8	81.3	79.2	−160.4	−70.3	−159.5
PNA1 AVG tot	−88.8	145.8	85.5	81.8	−161.1	−70.9	−158.7

^a^Calculated on the average of the 10 NMR structures of PDB structure 176D; ^b^from [40]; ^c^from [41]; ^d^from [42].
